# Social Inequality and Solidarity in Times of COVID-19

**DOI:** 10.3390/ijerph18126339

**Published:** 2021-06-11

**Authors:** F. Marijn Stok, Michèlle Bal, Mara A. Yerkes, John B. F. de Wit

**Affiliations:** Department of Interdisciplinary Social Science, Utrecht University, 3584 CH Utrecht, The Netherlands; m.bal@uu.nl (M.B.); m.a.yerkes@uu.nl (M.A.Y.); j.dewit@uu.nl (J.B.F.d.W.)

**Keywords:** COVID-19, social determinants of health, health inequalities, solidarity, social justice, intergenerational solidarity, global solidarity

## Abstract

The enormous public health burdens of the COVID-19 pandemic are not distributed equally. Inequalities are noticeable along socio-economic and socio-cultural fault lines. These social determinants of health affect both the prevalence and severity of COVID-19 infections as well as the magnitude of negative impacts of the measures taken to slow the spread of the virus. This perspective paper summarizes key inequalities in who is affected by SARS-CoV-2 infection and in who is affected by COVID-19 prevention measures, based on evidence presented in state-of-the-art literature, and discusses the scope of challenges that these inequalities pose to solidarity and social justice. Key challenges for solidarity are highlighted across three areas: challenges to intergenerational solidarity, to global solidarity, and to intergroup solidarity.

## 1. Introduction

COVID-19 (coronavirus disease 2019) is caused by SARS-CoV-2 (severe acute respiratory syndrome coronavirus 2), and symptoms include fever, cough, difficulty breathing, and fatigue [1]. SARS-CoV-2 spreads via close contact between people through small droplets [1]. While most people who acquire COVID-19 experience no or mild symptoms, a minority of (mainly elderly) people experience severe symptoms (e.g., dyspnea, hypoxia, respiratory failure, or multiorgan dysfunction) [1]. The World Health Organization declared COVID-19 a global pandemic on 11 March 2020. As of 6 June 2021, 172 million people have been diagnosed with COVID-19 across 217 countries, including more than 3.7 million people who have died [2]. The COVID-19 pandemic has developed into one of the most challenging public health crises in recent global history [3,4]. The enormous public health impact of COVID-19 directly results from its adverse health consequences and indirectly stems from the impact of the pandemic on the health system, as insufficient resources may be available to meet all needs, and essential health services may be redirected to the care for people affected by COVID-19, resulting in less and delayed care for adults and children with other serious conditions [5,6]. Moreover, COVID-19 prevention measures have negative economic and social consequences that are important in their own right and compound the public health impact of the pandemic [7,8,9]. Measures promoting social distancing, such as stay-at-home orders; closing or limiting access to schools, shops, restaurants, and public venues; cancelling large events and gatherings; restricting travel; and limiting the number of social contacts that people are advised to have, are especially disruptive to society with respect to their impact on the economy and collective wellbeing [8,9].

As such, the COVID-19 pandemic poses a worldwide systemic shock (i.e., having a tremendous and unexpected impact across public health systems, economies, and societies). This systemic shock was initially hailed by some journalists as “the great equalizer” [10]. The idea was that a virus does not discriminate, and hence COVID-19 would challenge all individuals and societies in similar ways. However, emerging evidence suggests otherwise. As with previous global pandemics [11], the burdens of the COVID-19 pandemic are not distributed equally. Inequalities are noticeable along many socio-economic and socio-cultural fault lines, referred to as the social determinants of health. The social determinants of health are “the conditions in which people are born, grow, live, work, and age” and are driven by “inequities in power, money, and resources” [12]. Social determinants of health affect both the prevalence and severity of COVID-19 infections as well as the magnitude of negative impacts of the measures taken to slow the spread of the virus [13]. These social inequalities in the impact of the COVID-19 pandemic are seen both within and between communities, countries, and global regions [4,14,15]. Social inequalities created or exacerbated by the COVID-19 pandemic raise many questions about solidarity and social justice. Understanding these social inequalities and the challenges that these inequalities pose to solidarity and social justice is the focus of this perspective paper. A clear overview of the various inequalities and improved insight into the resulting challenges to solidarity will facilitate further study, for example, into the potential effectiveness of various countries’ current responses as well as responses to future pandemics. After discussing the inequalities in who is affected by SARS-CoV-2 infection as well as inequalities in who is affected by COVID-19 prevention measures, we will highlight key challenges for solidarity and social justice that arise from these inequalities.

## 2. Inequalities in Prevalence and Severity of COVID-19 Infection

There is a strong relationship between COVID-19-related health outcomes (i.e., morbidity and mortality) and social determinants of health [16]. Specifically, ethnic and racial groups as well as socio-economically disadvantaged groups have been shown to bear a disproportionate share of the disease burden [17]. In the U.S., for example, over 33 million people have become infected with COVID-19 and nearly 600,000 have died, resulting in a mortality rate of 182 people per 100,000 inhabitants, according to Johns Hopkins University [18]. Crucially, it has been determined that these infection and mortality rates are about three times higher for African Americans than for European Americans [19], and mortality rates in the US have been shown to be nearly twice as high in high-poverty counties than in low-poverty counties [20]. Similar trends are observed around the world, both within and between countries. For instance, in Sweden, a high-income country, the overall mortality rate is estimated at 141 people per 100,000 inhabitants [18]—a much lower mortality rate than estimated in, for example, Brazil, a low-to-middle-income country where the morality rate is estimated at 223 people per 100,000 inhabitants [18]. Yet, within both countries, substantial differences are also observed. For example, COVID-19 infection rates in Sweden [21] are three to four times higher in some socioeconomically disadvantaged areas, and higher COVID-19 mortality has been reported in disadvantaged neighbourhoods in parts of Brazil [22].

There are several reasons for these social inequalities in the impact of COVID-19 (see Table 1). First, racial and ethnic minorities and socioeconomically disadvantaged communities are more likely to experience pre-existing, chronic health conditions, such as high blood pressure, heart conditions, and being overweight, which put them at higher risk of COVID-19-associated morbidity and mortality [17]. The higher prevalence of chronic conditions among these socio-economically disadvantaged groups is itself a result of systemic and enduring structural social inequity caused by unequal distributions of resources, power, and money [12,14]. Second, these same structural inequities make it more difficult for people in socially disadvantaged conditions to properly rest, exercise sufficiently, eat healthily, and avoid and navigate stress, which are all important health behaviours that contribute to boosting the immune system and mitigating the effects of COVID-19 [21,23]. Third, socio-economically disadvantaged population groups typically have lower health literacy and less access to correct health information, which makes them more vulnerable to misinformation and misperceptions [16,17,24]. As a result, socially disadvantaged people are less likely to adhere to preventive and protective measures, increasing their risk of acquiring SARS-CoV-2. Fourth, those who are less well-off typically have poorer health care insurance and health care access (including via telemedicine [25]) and receive less-optimal health care when they do present in a health care facility [23,26,27,28]. Socio-economically disadvantaged groups are thus less likely to receive optimal treatment if and when they become infected. Lastly, socially disadvantaged groups are likely less able to follow all prevention measures, resulting in higher risk of infection. Notably, lower-income workers and workers with lower education levels, for example, are less often able to work from home [29,30] and are often less able to socially distance during their work, due to the nature of their work (which is often in service sectors like hospitality, transportation, and health care [21]). Research has also shown that, despite equal willingness, people with lower incomes and people from black and minority ethnic groups feel less able to self-isolate than white people [30]. Furthermore, recommended preventive measures often assume that people have a home and (easy) access to running water and soap, which is not necessarily the case for specific groups of socially disadvantaged people [31], such as homeless people [32] and people living in close congregation [33], such as in slums [34], refugee camps [35], and prisons [36].

Another issue is that of severe inequity in access to COVID-19 vaccinations [37]. As it currently stands, there will be a time lag of at least 1.5 years between the time when many rich countries achieve widespread vaccination coverage and the time when the lowest-income countries do so [38]. High-income countries have established bilateral agreements with pharmaceutical companies early on and are initially focused on ensuring sufficient supply of vaccines for their own population. From an ethical point of view, it can be argued that—contrary to what is currently happening—vaccines should actually first be delivered to those countries where the health care system is least developed, as the risks of contracting COVID-19 are highest for people living in these countries [39]. Besides being ethically questionable, vaccine nationalism (where high-income countries’ primary focus is on securing swift vaccination of their own population [40,41]) also potentially exacerbates and prolongs the pandemic for the whole world, as the risk of virus mutations increases the longer the virus can freely continue to spread in certain parts of the world [40,41]. While there is a globally supported initiative that aims to ensure worldwide equitable access to vaccines (COVAX, the COVID-19 Vaccines Global Access Facility [42]), to which many high-income countries contribute, this initiative can only deliver enough vaccines for about 20% of the population of low-and-middle-income countries in 2021 [37]. Also, vaccine availability is only a first hurdle for low-and-middle-income countries; they also need to put substantial infrastructure in place (e.g., trained personnel, supply chains, appropriate storage facilities) to effectively deliver these vaccines to their populations [37,39]. Furthermore, within countries, various social interest groups, including professional societies, trade unions, and patient groups, lobby for earlier access to vaccination [43,44]. Instances of abuse of position have also been documented, with those in positions of power, wealth, or influence cutting the line to ensure vaccination for themselves and sometimes their family members or friends [43].

## 3. Inequalities in the Impact of COVID-19 Prevention Measures

Prevention measures are put in place to curb the spread of COVID-19, to protect the most vulnerable people, and to limit hospitalizations of people with COVID-19 as much as possible [8,9]. Yet, these preventive measures, especially those that promote social distancing, are themselves not without negative consequences (see Table 2). Several population groups are at a heightened risk of adverse consequences from social distancing measures.

First, while social distancing measures are likely to cause increased population-wide experiences of social isolation and psychological distress [45] and stress-related physical illness symptoms [46], negative mental health consequences appear to be higher for socio-economically disadvantaged groups [47,48,49]. Options for accessing both formal mental health services and receiving more informal mental support are reduced due to social distancing measures [49,50,51]. Many mental health care providers switched to online treatment (typically referred to as telepsychotherapy or telepsychiatry) during the pandemic. Yet, this type of online treatment seems less suitable for individuals with lower socio-economic positions and in lower-income societies [52,53,54]. Online mental health treatment requires access to a mobile device or computer, a stable internet connection, sufficient digital literacy, and access to a quiet and private place from which to participate in the online sessions [53]. It also requires training of mental health professionals to deliver treatment virtually, the development of consensus guidelines, the implementation of legal and ethical frameworks, and effective quality monitoring [52,53]. In addition, social distancing measures may have augmented negative consequences for those with pre-existing mental health conditions [50,55,56], which occur disproportionately among people with lower socioeconomic status. Pre-existing mental health conditions may exacerbate the negative effects and psychological distress experienced due to the pandemic and the social distancing measures, which cause large disruptions in daily activities and opportunities to receive support.

Second, many people face unfavourable economic consequences (loss of job, working hours, income) as a result of social distancing measures, with some sectors (e.g., tourism, hospitality) almost completely shut down for prolonged periods of time in many countries [7]. Affected workers are disproportionately female, young, low-paid, and on temporary contracts [29,57]. The resulting financial insecurity disproportionately harms those in lower socioeconomic groups [23], despite widespread economic support across the globe [58]. Indeed, it has been shown that economic inequity has grown staunchly since the beginning of the COVID-19 pandemic [7,59]. While billionaires’ wealth has continued to increase since the start of the pandemic, it is estimated that it will take more than a decade for the world’s poorest people to recover from the pandemic’s economic setbacks [59].

Third, social distancing measures appear to affect adolescents and young adults to a larger extent than any other age group [60,61]. Adolescence and young adulthood are phases of life in which social identities are formed and peers become the most important source of social influence [62]. The need for social connectedness is at its highest in this stage of life, and limiting young people’s ability to go out into the world and meet each other significantly impacts their wellbeing [63]. It has been shown that mental health problems, while significantly increasing across all population groups, grew most steeply among young adults [45,63]. Young people are thus asked to make large sacrifices to curb a disease that is typically not directly dangerous to them.

Fourth, a meta-analysis has shown that almost 80%of children across the world have been negatively affected by the pandemic and the implemented social distancing measures, including experiences of anxiety (35%), depression (42%), irritability (42%), boredom (35%), sleep disturbances (21%), excessive fear (23%), and inattention (31%) [64]. In addition, home violence against children and child abuse have increased during the pandemic [65]. The United Nations Educational, Scientific, and Cultural Organization [66] estimates that as part of social distancing measures, schools in at least 140 countries have experienced closures, affecting the education of 80% of all children worldwide. While likely negatively affecting the wellbeing of most children, these measures disparately affect already vulnerable children. Children from socioeconomically disadvantaged backgrounds often rely on school meal programmes [67,68]. Furthermore, schools can provide a safe space and access to safe, trusted adults for children in unstable home environments [33,68], which is taken away during a school closure. Moreover, while it is likely that school closures may negatively affect educational outcomes and academic performance across the board, these effects are likely to be much larger among children from socioeconomically disadvantaged backgrounds, who are more likely to live in situations that make home schooling difficult (e.g., no quiet place to do homework, no stable Internet connection, no books available, no adequate heating, less help available [67,69]). This situation has exacerbated the gap in academic achievements between lower and higher socioeconomic background children [70].

Fifth, and relatedly, school closures place a large burden on many parents [29,71]. A meta-analysis [64] has shown that a substantial number of parents developed negative psychological symptoms during school closures and social distancing, with 52% reporting experiences of anxiety and 27% reporting symptoms of depression. Parents are required to take care of their children at home, take on responsibility for home schooling, and manage their work requirements as well as attend to any other obligations. Parents thus face a substantial additional demand on their time, while due to social distancing measures, outside help (e.g., from grandparents or babysitters) is less accessible. This situation leads to increases in perceived work pressure and stress (that are not distributed equally across men and women [72]). The burden is even higher on poorer families, who are less likely to be able to work from home, less able to take days off of work to take care of their children, and have lower financial resources to cover unforeseen expenditures [29,72].

## 4. Challenges to Solidarity and Social Justice in the Ongoing Pandemic

The social inequalities in both the health impacts of the virus and the consequences of preventative measures taken to curb the spread of the virus uniquely challenge solidarity and social justice. Initially, the response to the crisis was one of empathy and solidarity. Many governments stressed we are all in this together and that we could only get out of the pandemic by staying together and being solidaristic (e.g., the Dutch government began a national campaign entitled “Only together will we get the coronavirus under control” (“Alleen samen krijgen we corona onder controle”), and the USA had the #AloneTogether campaign). This call for solidarity resonated with the public, and initiatives were developed to support vulnerable population groups, such as the elderly (including those highlighted by the municipality of Amsterdam [73]; see also [74,75,76]), and express appreciation of the critical contributions of essential workers [77,78]. However, as the pandemic continues, solidarity and social justice could be increasingly under pressure [77,79].

The pandemic presents at least three immediate challenges for solidarity [80] (see Table 3): how to balance upward and downward solidarity between generations, global solidarity across high-versus-low and middle-income countries (LIMC), and intergroup solidarity between persons with and without an Asian appearance, and the potential stigma attached to having an Asian appearance.

First, a key challenge in the pandemic relates to intergenerational solidarity, that is, solidarity between generations. COVID-19 was initially presented mainly as a threat to the elderly [81,82,83,84]. For this reason, younger generations were asked to be solidaristic with older generations (i.e., upward solidarity [85]). Social scientists noted many solidaristic initiatives to support the vulnerable elderly, for example, by helping them buy groceries or by checking in with them to reduce loneliness [81,82]. (It should be noted that because the virus was framed as primarily affecting older individuals, some scholars warned against increases in ageism and called for action against ageist discourse [81,82,83,84]. They referred to harsh social media hashtags, such as “#BoomerRemover”, harmful quotes by politicians, e.g., stressing the economic benefits of the pandemic affecting older persons, and ongoing public debates on triage criteria, i.e., the priority assigned to patients presenting with coronavirus, reflecting blatant ageist attitudes). Yet, as the pandemic continues, and with young people more negatively affected by social distancing measures, there is a shift from upward to downward expectations of intergenerational solidarity in public discourse [86,87,88,89]. Younger people (and/or organizations speaking on their behalf) are now asking for solidarity from the elderly to make it possible to lessen some social distancing measures (e.g., by proposing the elderly stay home to protect themselves), thereby reducing some of the harmful social consequences of these measures that are particularly detrimental to younger generations. In other words, the COVID-19 pandemic and preventive measures are resulting in appeals for reciprocity in intergenerational solidarity. Younger generations (or others on behalf of them) are now asking older generations to stay at home in return for their earlier adherence to the social distancing measures to protect the elderly.

Previous research has shown that, whereas formal solidarity in general is mainly directed upward (e.g., through pension systems and institutionalized old age care), informal solidarity is more often directed downward, with intergenerational transfers of money and time being provided by older generations to younger ones within families (e.g., through caring for grandchildren and providing financial support for buying a (first) house [85,90]). Overall, transfers of money and time seem to even out between generations when taking into account both formal and informal forms of solidarity [91]. However, in the current pandemic, it may be even more difficult to balance upward and downward solidarity, as the commodities having to be balanced are not just money and time. Questions about what is a fair balance and what one can reasonably expect from other generations are difficult to answer. In terms of distributive justice, i.e., the fair allocation of burdens and benefits [92], how can one equate the availability of hospital beds with the value of mental well-being? Another factor further complicating solidarity is that some groups in societies (oftentimes older age groups) receive vaccines earlier than others. This raises additional questions about who is deserving of what in terms of loosening social distancing measures and travel bans (e.g., loosening measures for vaccinated groups to no longer impede on their well-being vs. staying solidaristic with the groups who have not had the opportunity to receive a vaccine yet). Taken together, the COVID-19 pandemic challenges intergenerational solidarity, raising new questions regarding the fair allocation of burdens and benefits.

A second key challenge relates to global solidarity, with the scope of social justice stretched to geographical boundaries. In this global pandemic, people are expected to be solidaristic with others across the globe, disadvantaged populations in particular. At the same time, the threat and uncertainty of the COVID-19 pandemic may push people towards protecting their own group rather than being solidaristic with others worldwide [93]. As such, some scholars question whether such global solidarity is possible in an age of growing populist nationalism [94]. In two U.S.-based experimental studies, support was indeed found for a reduction in global solidarity. Both exposure to the effects of COVID-19 [95] as well as concern about the impact of COVID-19 on their country’s financial situation [96] reduced people’s support for development assistance. However, these studies also showed that global solidarity increased when national benefits of providing development assistance were emphasized (e.g., when developmental support could help curb the next wave of the disease at home). In a German sample [97], public support for development assistance was not influenced negatively by threat perceptions. In fact, in people who trust their government, concerns about the loss of friends or relatives even increased support for development assistance.

This does raise the relevant question of how public support for global solidarity develops if levels of trust in the government decrease. Levels of trust in the government—as well as the effects of threat perceptions—may change over the course of the pandemic. Studies thus far focused on global solidarity in the first phase of the pandemic. Now that the pandemic has been ongoing for over a year, global solidarity is increasingly challenged [77,79]. For instance, fractures in solidarity are revealed with the development of vaccines that are being bought up by high-income countries at the expense of low- and middle-income countries [37,40,41]. This vaccine nationalism is a threat to the COVAX [42] program that aims to ensure access for low- and middle-income countries [94] and, with that, to global solidarity. Moreover, vaccination rates are rapidly increasing, at least in some, mostly high-income, countries, and several social distancing restrictions have been or can be loosened in the near future here. Life going back to normal in high-income countries leads to the risk that solidarity with people and regions lagging behind in vaccination access will decrease.

By identifying on the global level, people, and especially those in high-income countries, may feel they are exposed to similar risks from the virus compared to people in developing countries and may also experience similar burdens related to social distancing measures taken to slow the spread of the virus. Moreover, this global identity may enable a shift in our justice principles from the Western default equity perspective (emphasizing proportionality) to the more solidaristic and caring principles of equality and need [98]. While the former is focused on whether people have contributed enough to warrant a pay-off (e.g., whether societies have done enough to gain access to vaccines), the latter are focused more on the macro-perspective of creating a fair world (e.g., how vaccines need to be distributed to end the pandemic as soon as possible). As such, emphasizing and stimulating a global human identity and with that, increasing global solidarity, will be a key challenge towards finding the quickest way out of the pandemic.

A third key challenge relates to intergroup solidarity. Intergroup solidarity is challenged during the COVID-19 pandemic through the emergence of new forms of stigma [99,100]. Stigmatization in the context of infectious diseases can be observed throughout history and directed at various groups, for example, toward African people in relation to Ebola outbreaks [101] and towards Latinos during the H1N1 influenza pandemic [102]. In the case of the COVID-19 pandemic, stigma is directed towards people with an Asian appearance because the virus originated in China. An increase in discriminatory behaviours, including acts of physical violence, towards people with an Asian appearance has been observed worldwide [99,100]. Stigmatization affects the mental health and wellbeing of people experiencing stigma and makes disease control more complex, as people experiencing stigma might be hesitant to seek health care assistance.

## 5. Conclusions

COVID-19 will continue to affect individuals, communities, and societies worldwide for some time to come. We are currently experiencing the immediate effects of the pandemic in terms of morbidity and mortality, societal well-being, and exacerbated social inequalities, most notably with regard to socio-economic position. From this perspective, we reviewed these direct consequences and their implications for solidarity and social justice, providing the field with an overview of the various ways in which the pandemic affects different population groups. We showed that the pandemic is not the great equalizer some expected it to be [10]. The pandemic affects socioeconomically disadvantaged population groups and countries more than others. In addition, social distancing measures affect population groups unequally. Specific occupational sectors, young people, women, people with pre-existing (mental) health conditions, and lower socioeconomic population groups are affected most. The pandemic and preventive responses also challenge solidarity both intergenerationally, globally, and between population groups. Our synthesis of these challenges can provide a basis for critical appraisal of the current situation, comparison of different existing policy responses, and may point to potential entry points for effective policy responses now and in the future. How societies rise to these new challenges for solidarity and social justice will only become fully clear over time, but their responses may significantly alter our future.

## Figures and Tables

**Table 1:** Schematic overview of reasons for social inequalities in impact of COVID-19.

Reason for Inequality	Brief Explanation
Pre-existing health conditions	Socioeconomically disadvantaged communities and racial and ethnic minority groups are more likely to experience pre-existing, chronic health conditions that put them at higher risk of COVID-19-associated morbidity and mortality.
Fewer opportunities for supporting immune system	Socioeconomically disadvantaged communities and racial and ethnic minority groups typically have fewer opportunities to rest, exercise, eat healthily, and avoid and navigate stress, which all boost the immune system which can help mitigate effects of COVID-19.
Lower health literacy	Socioeconomically disadvantaged communities and racial and ethnic minority groups typically have lower health literacy, which makes them more vulnerable to misinformation and less likely to follow all preventive and protective measures.
Suboptimal health care (access)	Socioeconomically disadvantaged communities and racial and ethnic minority groups more often have suboptimal health care insurance and health care access and more often receive suboptimal health care.
Less opportunity to follow preventive and protective measures	It is often more difficult for people from socioeconomically disadvantaged communities and from racial and ethnic minority groups to, e.g., work from home, socially distance during work, self-isolate, and observe hygienic prevention measures.

**Table 2:** Schematic overview of inequalities in the impact of COVID-19 prevention measures.

Type of Impact	Brief Explanation of Inequality
Negative mental health consequences	Negative mental health consequences appear to be higher for socioeconomically disadvantaged groups both because social distancing measures reduce access to mental health services and informal mental support more for people from socioeconomically disadvantaged groups than for people from advantaged groups, and because pre-existing mental health conditions, which can augment negative mental health consequences, occur more frequently in disadvantaged groups.
Unfavorable economic consequences	Societal shutdowns (e.g., in hospitality and tourism) have had a disproportionately negative effect on the financial security of those in lower socio-economic groups, and economic inequity has grown staunchly during the pandemic.
Wellbeing of adolescents and young adults	Social distancing measures disproportionately burden young people for whom the need for social connectedness is often augmented compared to older adults, while the disease is typically not directly dangerous to them.
Wellbeing of children, especially socioeconomically disadvantaged children	A majority of the world’s children have been negatively affected by social distancing measures, for example, due to school closures. These closures disparately affect children who were already socioeconomically disadvantaged.
Wellbeing of parents, especially socioeconomically disadvantaged parents	Social distancing measures place excessive additional burdens on parents, leading to increased work pressure and stress; these burdens are disparately experienced by parents in poorer socio-economic households and, in many countries, women more than men.

**Table 3:** Schematic overview of challenges to solidarity arising from the COVID-19 pandemic.

Challenge	Brief Explanation
Intergenerational solidarity	A fair intergenerational allocation of burdens and benefits in the COVID-19 crisis seems complex and challenging. Younger generations were initially asked to be solidaristic with older generations by adopting social distancing and other preventive measures; in later stages of the pandemic, younger people have called upon older generations to support lessening of some preventive measures to reduce detrimental effects for younger generations.
Global solidarity	Solidarity with others across the globe, especially disadvantaged populations, is a challenge during the COVID-19 crisis. People are expected to be solidaristic with those who are less well-off, but the threat and uncertainty of the pandemic may push people towards protecting their own group. Vaccine allocation and distribution is a topic that warrants special attention in this regard.
Intergroup solidarity	New forms of stigma have developed during the COVID-19 pandemic, challenging intergroup solidarity. Stigmatization affects the mental health and wellbeing of people experiencing stigma and makes disease control more complex.

## Data Availability

Not applicable.
